# A unique uterine cervical “teratocarcinosarcoma”: a case report

**DOI:** 10.1186/s13000-019-0890-5

**Published:** 2019-11-04

**Authors:** Kozue Ito, Mitsutake Yano, Aiko Ogasawara, Maiko Miwa, Eito Kozawa, Masanori Yasuda

**Affiliations:** 1 Department of Pathology, Saitama Medical University International Medical Center, 1397-1 Yamane, Hidaka-City, Saitama, 350-1298 Japan; 2 Department of Obstetrics and Gynecology, Oita University Faculty of Medicine, Yufu-shi, Oita 879-5593 Japan; 3 Department of Gynecologic Oncology, Saitama Medical University International Medical Center, 1397-1 Yamane, Hidaka-City, Saitama, 350-1298 Japan; 4 Department of Diagnostic Radiology, Saitama Medical University Hospital, Moroyama, Saitama, 350-0495 Japan

**Keywords:** Uterine cervix, Teratoma, Carcinosarcoma, Teratocarcinosarcoma

## Abstract

**Background:**

Teratocarcinosarcoma (TCS) is a rare aggressive tumor of the nasal cavity and paranasal sinuses and has both epithelial and two or more mesenchymal components. In other organs, 5 cases of ovarian tumors closely resembling TCS have been reported; however, there has been no published case of cervical TCS. Herein, we describe a unique case of cervical tumor that had carcinosarcomatous and teratomatous features, resembling a sinonasal TCS.

**Case presentation:**

A 45-year-old woman presented to our hospital for evaluation of a cervical lesion. The gynecologist found a large polypoid mass, whose biopsy showed glandular components of probable germ cell origin based on the immunohistochemical features. The patient underwent total hysterectomy with bilateral salpingo-oophorectomy. The cervical polypoid mass was found to consist of both epithelial and mesenchymal tissues, including immature glandular structure resembling fetal enteric tubules, neuroepithelial cells, hyaline cartilage, and rhabdomyosarcoma cells. This tumor was diagnosed as TCS of the uterine cervix. Following the surgery, the patient received radiotherapy and has been free of disease for 13 months.

**Conclusion:**

This is the first case report of cervical TCS. The tumor is thought to be histogenetically less associated with HPV infection, and its teratomatous components with an absence of cytogenetic abnormalities (including isochromosome 12p (i(12p)) suggest a analogous histogenesis compared to pure mature or immature teratoma.

## Background

Carcinosarcoma [1, 2] and teratoma [3, 4] are encountered in the uterine cervix, although rarely. Cervical carcinosarcoma, characterized by a mixture of both malignant epithelial and mesenchymal elements, is an aggressive neoplasm with poor prognosis [1, 2]. Teratomas consist of two or three germ layers: ectodermal, mesodermal, and endodermal components in any combination. Mature teratoma is usually benign, but 1–3% cases can show malignant transformation; immature teratoma consists of embryonic tissue, mainly neural tissue. Cervical teratoma is thought to develop from germ cells that get trapped along their migration during embryonic development [3, 5].

Teratocarcinosarcoma (TCS) has both epithelial components and two or more mesenchymal components, such as cartilage, fibroblasts, muscular, and bony tissues. The tumor, known to arise in the nasal cavity and paranasal sinuses, was first described as a teratoid carcinosarcoma by Shanmugaratnam et al. in 1983 [6], whereas the term TCS was initially proposed by Heffner and Hyamsin in 1984 [7]. Since then, there have been less than 100 cases with tumors originating from the nasal cavity or paranasal sinuses reported in the English literature [8]. The patients were predominantly male (87%) with an average age of 54.5 years [7, 8]. TCS has several clinicopathological features and problems, such as poor prognosis due to rapid growth, many differential diagnoses, and ambiguities of histogenesis or tumor origin. In sinonasal TCS, radical surgery followed by radiotherapy is the most commonly performed treatment option, however, resulting in a poor prognosis (average survival, 1.7 years) due to frequent recurrence and metastasis [7, 8]. Because TCS shows histologically diverse features such as mature/benign as well as immature / malignant epithelial and mesenchymal components, pathologists need to distinguish TCS from carcinosarcoma, immature teratoma, teratoma with malignant transformation, and peripheral primitive neuroectodermal tumor. TCS has been reported to occur in the female reproductive organs, especially in the ovary, other than in the nasal cavity and paranasal sinuses [9–13]. Ovarian TCS is thought to originate from germ cells [9–13].

Herein, we report a unique uterine cervical tumor with carcinosarcomatous and teratomatous features, histologically resembling sinonasal TCS.

## Case presentation

### Clinical history

**Fig. 1:**
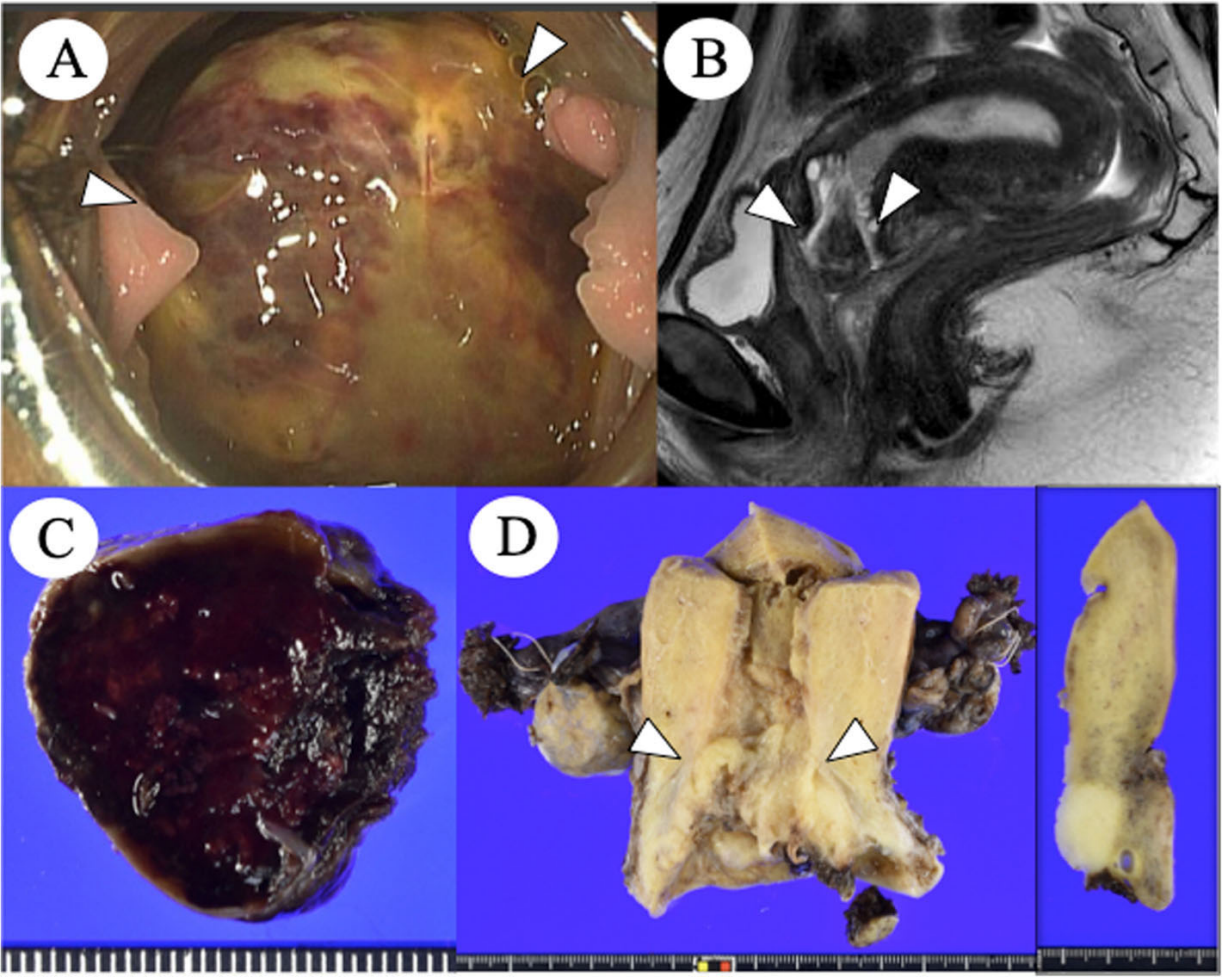
**a** Colposcopic image of the cervical mass (arrowhead). **b** Sagital magnetic resonance image of the low-intensity cervical mass (arrowhead). **c** Cut section of the cervical mass. **d** Yellow-white surgical specimen (arrowhead) from the uterus. Inset: cut surface of the uterus

A 45-year-old Japanese woman, gravida 2, para 2, with no remarkable medical history, presented to our hospital for evaluation of a cervical lesion. At her first visit, a gynecologist was not able to detect a mass, but 3 weeks later, the gynecologist found a large polypoid mass on the cervix (Fig. 1a). The patient’s laboratory data, including serum tumor markers (carbohydrate antigen 125, alpha-fetoprotein, human chorionic gonadotropin, carbohydrate antigen 19–9, and carcinoembryonic antigen), were within normal limits. Magnetic resonance imaging revealed a mass on the cervix measuring 28 × 27 × 20 mm (Fig. 1b), with no contrast enhancement. ^18^F-fluorodeoxy-D-glucosepositron emission tomography revealed an abnormal uptake in the polyp and no significant findings on the uterine corpus, bilateral adnexa, and other organs. The polypectomy specimen histologically suggested a malignant germ cell tumor. Subsequently, the patient underwent total abdominal hysterectomy with bilateral salpingo-oophorectomy. Following surgery, she received radiotherapy and has been free of disease for 13 months.

### Pathologic findings

#### Macroscopic findings

The cervical polyp was dark in color and pedunculated, measuring 33 × 30 × 25 mm, with a red-tan cut surface due to massive hemorrhage (Fig. 1c). The hysterectomy specimen revealed the cervix almost entirely occupied by a yellow-white solid tumor, measuring 45 × 40 × 17 mm (Fig. 1d).

#### Microscopic findings

**Fig. 2:**
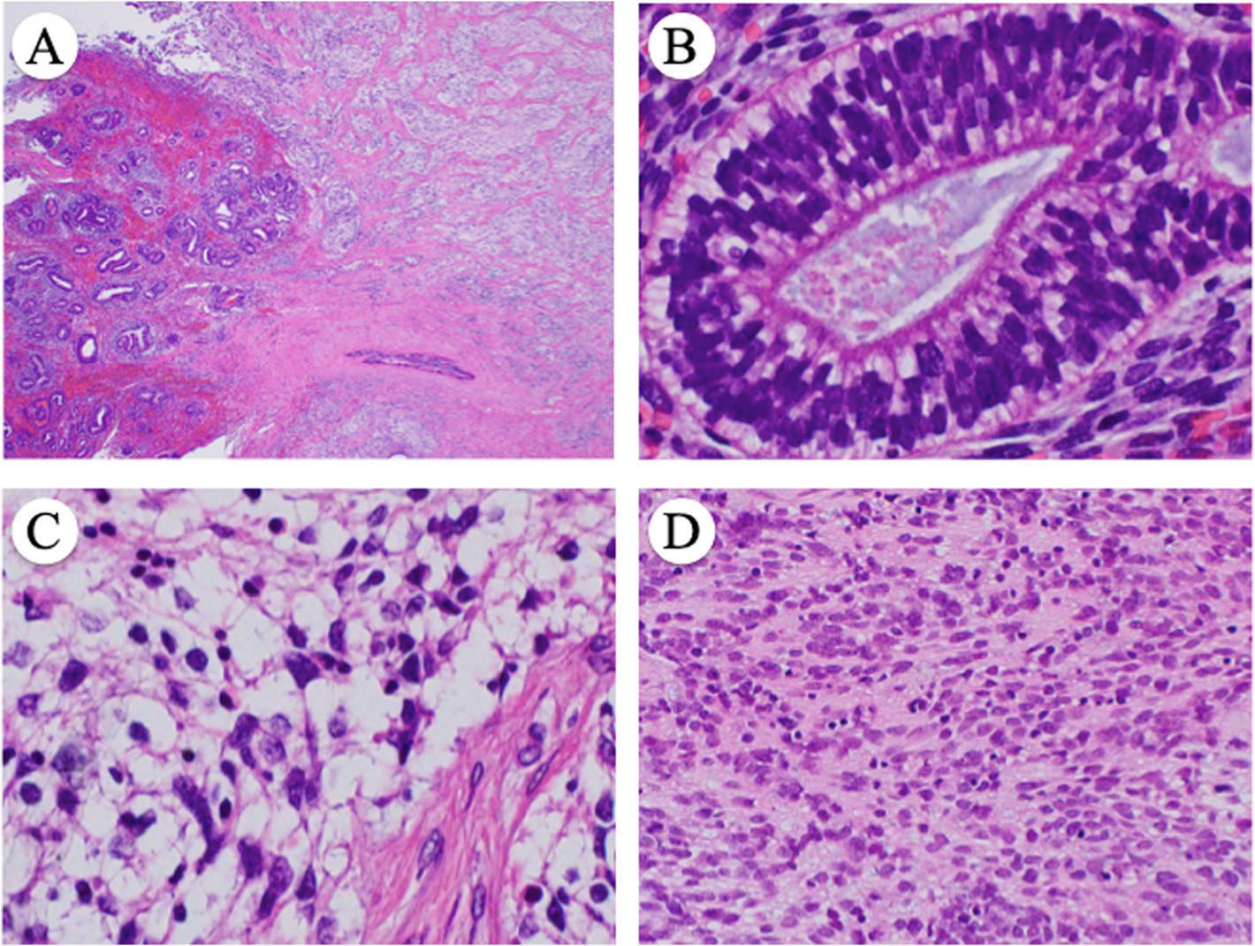
**a** Abrupt transition between carcinosarcomatous (left) and neuroectodermal (right) components (magnification × 100). **b** Immature epithelial gland (magnification × 400). **c** Neuroectodermal tissue (magnification × 400). **d** Small blue cells with neuroendocrine differentiation (magnification × 200)

**Fig. 3:**
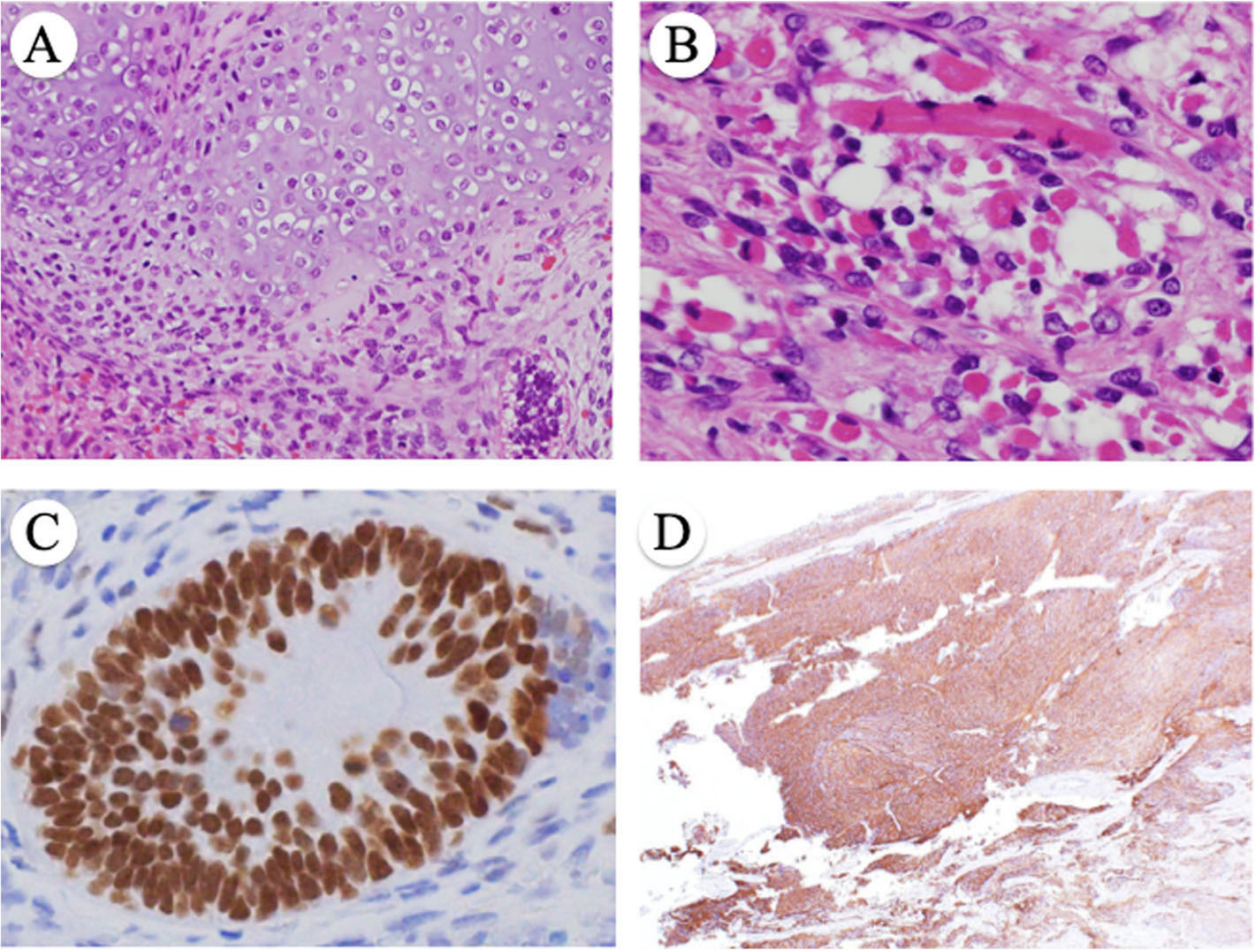
**a** Hyaline cartilage (magnification × 200). **b** Striated muscle (magnification × 400). Immunostained images of (**c**) SALL4 (magnification × 400) and (**d**) synaptophysin (magnification × 200)

The cervical tumor showed both epithelial and mesenchymal tissues consisting of immature glandular cells (Fig. 2b), neuroepithelial tissue (Fig. 2c), small blue cells with neuroendocrine differentiation (Fig. 2d), hyaline cartilage (Fig. 3a), and rhabdomyosarcoma (Fig. 3b). Among these tissues, the most prominent component was neuroepithelium, and the tumor contained no immature squamous epithelium, which is common in sinonasal TCS. The tumor had invaded more than half the cervical stroma, but with no involvement of the parametrium or vagina. The tumor had prominent lymphovascular infiltration and extended to the endometrium. Bilateral fallopian tubes and ovaries were free of the tumor.

#### Immunohistochemical features

Table 1 shows a summary of the immunohistochemical staining. Immature glandular epithelium stained positive for Sal-like protein 4 (SALL4) (Fig. 3c), glypican-3, caudal-type homeobox transcription factor 2 (CDX-2) (focally), and CD99, but negative for octamer-binding transcription factor 4 (Oct-4) and estrogen receptor. The neuroepithelium stained positive for neuron-specific enolase, synaptophysin (Fig. 3d), and CD99, but negative for cytokeratin and Oct-4.

**Table 1:** Immunohistochemical profile of each component

Marker	Neuroendocrine Tissue	Glandular Epithelium	Stromal Tissue	Neuroepithelium
SALL4	+ (focal)	+	–	+ (focal)
Glypican-3	+ (focal)	+	–	+ (focal)
Oct-4	–	–	–	–
S-100	–	–	–	–
PLAP	–	–	–	–
NSE	+	–	–	+
Neurofilament	+ (focal)	–	–	–
GFAP	–	–	–	–
CD56	+	+	–	+
CD57	+	–	+	+
CD99	+	+	+	+
Synaptophysin	+	–	–	+
Chromogranin A	–	–	–	–
AFP	–	–	–	–
TTF-1	–	–	–	–
CDX-2	–	+ (focal)	–	–
WT1	–	+ (focal)	–	–
ER	–	–	–	–
CD10	–	–	–	–
PAX-8	+(focal)	+	+	+(focal)
β-catenin	–	+(membrane)	–	–
AE1/AE3	–	+	–	–
CD34	–	–	+	–
Desmin	–	–	+ in STMC	–
α-SMA	–	–	–	–
MyoD1	–	–	+ in STMC	–

*AFP*, Alpha-fetoprotein; *α-SMA*, Alpha-smooth muscle actin; *CDX-2*, Caudal-type homeobox transcription factor 2; *ER*, Estrogen receptor; *GFAP*, Glial fibrillary acidic protein; *NSE*, Neuron-specific enolase; *Oct-4*, Octamer-binding transcription factor 4; *PLAP*, Placental alkaline phosphatase; *SALL4*, Sal-like protein 4; *STMC*, Striated muscle cells; *TTF-1*, Thyroid transcription factor 1; *WT1*, Wilms’ tumor 1

#### Cytogenetic analysis with FISH

**Fig. 4:**
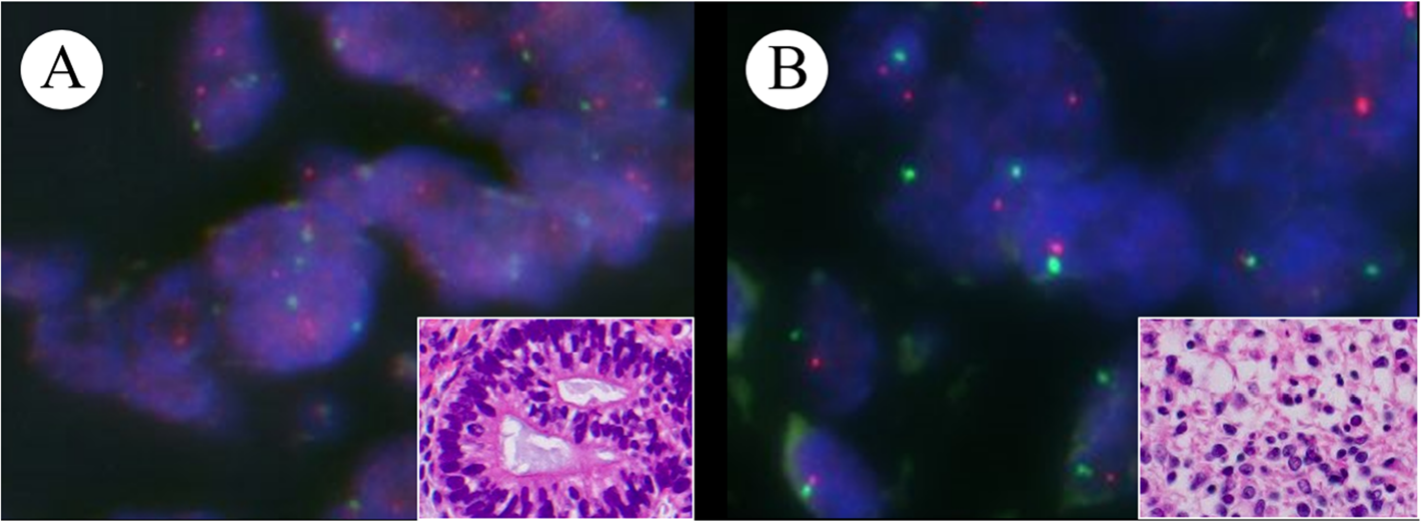
Chromosome 12p to centromere ratio is not increased by FISH (green signals-12p telomere; red signals-12 centromere): (**a**) in immature / atypical glands (inset, H&E stain), and (**b**) in neuroepithelium (inset, H&E stain)

Human papillomavirus (HPV) infection was not detected by in situ hybridization (GenPoint HPV; Dako), and no evidence of *ESWR1* gene rearrangement was detected by fluorescence in situ hybridization (FISH) (VysisLSIESWR1 Dual Color Break Apart Probe). Furthermore, neither isochromosome 12p (i(12p)) nor 12p amplification was observed by FISH (Cytocell 12pter Subtelomere Specific Probe / Cytocell Chromosome 12 Alfa Satellite Probe) (Fig. 4). Based on these findings, the tumor was diagnosed as TCS of the uterine cervix, FIGO stage IB2 (pT1b2 pNX cM0).

## Discussion

The present case is a unique cervical cancer, consisting of neuroepithelial components positive for CD99 and synaptophysin, immature / atypical intestinal glands positive for SALL4 and CDX-2, cartilage cells, and rhabdomyosarcoma, resembling sinonasal TCS. There have been less than 100 cases of TCS reported in the literature to date, most of which were observed to occur in the nasal cavity or paranasal sinuses [8], but were also in female reproductive organs, especially the ovary [9–13] (Table 2). TCS has several differential diagnoses such as carcinosarcoma, immature teratoma, teratoma with malignant transformation, and peripheral primitive neuroectodermal tumor, because of its histological heterogeneity.

**Table 2:** Teratocarcinosarcomas of the female genital tract

Author [year]	Age, y	Primary	Diagnosis	Treatment	Stage	Histology	Outcome
Ehrmann RL [1990]	62	Ovary	TCS	Surgery + chemotherapy	III	SCC, adenocarcinoma, chondrosarcoma, rhabdomyosarcoma	DOD 27 mo
Tanimoto A [2001]	59	Ovary	TCS	Surgery	IV	SCC, adenocarcinoma, chondrosarcoma	DOD
Garcia-Garvis OF [2008]	69	Ovary	TCS	Surgery	IV	SCC, adenocarcinoma, chondrosarcoma, rhabdomyosarcoma, osteosarcoma, liposarcoma, yolk-sac tumor	DOD
Matsuura Y [2010]	40	Ovary	TCS	Surgery + chemotherapy	II	Adenocarcinoma, chondrosarcoma, endometrial stromal sarcoma	DOD 46 mo
Coutney F [2019]	55	Ovary	TCS	Surgery + chemotherapy	IC	Adenocarcinoma, carcinosarcoma, dysgerminoma, primitive retinal tissue primitive neuroepithelial tissue, fetal cartilage	DOD 14 mo
Present case [2019]	45	Cervix	TCS	Surgery + radiation	IB2	Glial-like tissues, immature intestinal glands, cartilage, neuroepithelial tissue, rhabdomyosarcoma	NED 13 mo

*TCS*, Teratocarcinosarcoma; *SCC*, Squamous cell carcinoma; *DOD*, Died of disease; *NED*, No evidence of disease

Currently, teratomas of the ovary and cervix are thought to have a parthenogenetic origin from the oocyte after completion of the first division [5]. Extragonadal teratoma usually arises in midline structures, such as the sacrococcygeal, mediastinal, and sacral regions, and rarely arises in the uterine cervix [5]. Grayson et al. [2] reported that in 8 cases with uterine cervical carcinosarcoma, HPV was detected in all. However, the present case showed no HPV infection, which was confirmed by the negative reactions of immunohistochemical staining for p16^INK4a^ and HPV in situ hybridization. Immature teratoma is defined by the presence of neuroepithelial tubules and rosettes. In the present case, however, the tumor predominantly consisted of neuroepithelial tissues, but lacked neuroepithelial tubules or rosettes. In addition, the tumor was immunohistochemically negative for Oct-4, which is usually positive for immature teratoma grade 2/3 [14]. In this case, malignant transformation of mature teratoma could be ruled out because of an absence of continuity between malignant or immature components and mature teratomatous tissues. Peripheral primitive neuroectodermal tumor was considered unlikely because *ESWR1* rearrangement was not observed.

In the present case, immature / atypical intestinal glands and neuroepithelium were positive for SALL4, which is known as a sensitive and specific marker of germ cell tumors [15]. This suggests that this tumor is germ cell derived. In the pure mature or immature teratoma, there is the presence of i(12p) and 12p amplification in non-teratoma germ cell components in ovarian or sacrococcygeal tissues [16, 17]. However, this case showed neither i(12p) nor 12p amplification. The absence of cytogenetic abnormalities in this case suggests that the teratomatous components have an analogous histogenesis compared to the pure ovarian teratoma.

The histogenesis of cervical TCS remains to be clarified, but teratoid carcinosarcoma of the ovary has been reported to show components derived from germ cells [9–13]. Although germ cells are not usually found in the uterine cervix, extragonadal germ cell tumors rarely occur in the uterine cervix [3–5]. The cases of ovarian TCS showed a poor clinical outcome mainly because they were at the advanced stage. Radical surgical resection followed by radiotherapy is the most common treatment option for sinonasal TCS [7, 8]; therefore, a combination of surgery and radiotherapy may be expected to be effective for the present case with TCS.

## Conclusion

This is the first case report of uterine cervical TCS that may have originated from germ cells, but with a histogenetic pathway different from pure mature teratoma of the ovary.

## Data Availability

The datasets used and / or analyzed during the current study are available from the corresponding author upon reasonable request.

## Abbreviations

CDX-2: Caudal type homeobox transcription factor 2
FISH: Fluorescence in situ hybridization
HPV: Human papillomavirus
Oct-4: Octamer-binding transcription factor 4
SALL4: Sal-like protein 4
TCS: Teratocarcinosarcoma
